# A novel mutation in *CRYAA* is associated with autosomal dominant suture cataracts in a Chinese family

**Published:** 2012-12-26

**Authors:** Dongmei Su, Yuanyuan Guo, Qian Li, Lina Guan, Siquan Zhu, Xu Ma

**Affiliations:** 1 National Research Institute for Family Planning, Department of Genetics, Beiing 100730, China; 2 Capital Medical University,Beijing Ophthalmology & Visual Sciences Key Lab, Beijing, China; 3 WHO Collaborative Center for Research in Human Reproduction, Beiing, China

## Abstract

**Purpose:**

To identify the genetic defect in a three-generation Chinese family with congenital cataracts.

**Methods:**

The phenotype of a three-generation Chinese family with congenital cataracts was recruited. Detailed family history and clinical data of the family were recorded. Candidate gene sequencing was performed to screen out the disease-causing mutation. Bioinformatics analysis was performed to predict the function of the mutant gene.

**Results:**

The phenotype of the family was identified as Y-suture cataract by using slit-lamp photography. Direct sequencing revealed a c.161G>C transversion in exon 1 of crystallin, alpha A (*CRYAA*). This mutation cosegregated with all affected individuals in the family and was not found in unaffected family members or in the 100 unrelated controls. Bioinformatics analysis indicated that the 54th amino acid position was highly conserved and the mutation R54P caused an increase in local hydrophobicity around the substitution site.

**Conclusions:**

This study identified a novel disease-causing mutation c.161G>C (p.R54P) in *CRYAA* in a Chinese family with autosomal dominant Y-suture cataracts. This is the first report relating a G→C mutation in *CRYAA* leading to congenital Y-suture cataract.

## Introduction

Congenital cataract is the most common cause of treatable childhood blindness. Worldwide, more than 1 million blind children in Asia suffer from cataracts [1]. The cataract may be isolated, may be associated with other developmental abnormalities of the eye, or may form part of an inherited multisystem disorder. Approximately one-quarter to one-third of congenital cataracts is inherited and has been reported with all three types of Mendelian inheritance, including autosomal dominant, autosomal recessive, and X-linked. Most inherited cataracts manifest as an autosomal dominant trait in which penetrance is almost complete but expressivity is highly variable [2]. According to morphology, congenital cataracts can be classified into several subtypes: sutural, pulverulent, whole lens, nuclear, amellar, cortical, polar, cerulean, coralliform, and other minor subtypes [3]. These subtypes of cataracts can result from mutations at different genetic loci and can have different inheritance patterns.

Approximately half of all families with cataract have crystalline mutations, including crystallin, alpha A (*CRYAA*), crystallin, alpha B (*CRYAB*), crystallin, beta A1 (*CRYBA1/A3*), crystallin, beta B1 (*CRYBB1*) crystallin, beta B2 (*CRYBB2*), crystallin, gamma C (*CRYGC*), crystallin, gamma D (*CRYGD*), and crystallin, gamma S (*CRYGS*). About one-quarter have connexin mutations in gap junctional proteins, including gap junction protein alpha 3 (*GJA3*) and gap junction protein alpha 8 (*GJA8*), with the remainder divided among the genes for heat shock transcription factor-4 (*HSF4*), aquaporin-0 (AQP0 *MIP*), and beaded filament structural protein-2 (*BFSP2*) [4].

In this study, we applied a functional candidate approach to test the known genes in a Chinese family. A novel missense mutation in *CRYAA* was identified as responsible for the cataracts in the family.

## Methods

### Clinical examination and isolation of genomic DNA

**Figure 1:**
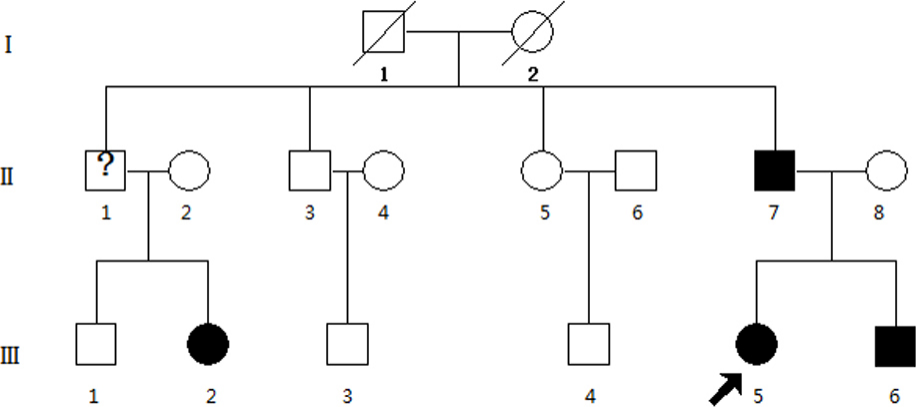
A Chinese family that has had autosomal dominant cataracts. The arrow indicates the proband.

A three-generation Chinese family from Hebei Province with autosomal dominant congenital cataract was examined at Beijing Tongren Hospital (Figure 1). One hundred unrelated subjects without eye diseases except mild myopia were also recruited from the Ophthalmology Clinic of Beijing Tongren Hospital as normal controls. The ethics committee of Capital Medical University approved the research, and all participants from the family gave informed consent. The study protocol followed the principles of the Declaration of Helsinki.

Six family members who were from Hebei Province (II:5, female, 45 years; II:7, male, 43 years; II:8, female, 43 years; III:4, male, 21 years; III:5, female,16 years; III:6, male, 13 years) participated in the study. All participating members were healthy when they were recruited, all of them underwent ophthalmic examination in Beijing Tongren Hospital. A history of cataract extraction or ophthalmologic examination was used to determine those whose status was considered to be affected; six family members (II:5, II:7, II:8, III:4, III:5, III:6) participated in the study. All participating members underwent ophthalmic examination, including visual acuity, slit-lamp examination, intraocular pressure measurement, ultrasonography, and fundus examination of the dilated pupil. The phenotypes were documented with slit-lamp photography, and 5 ml of venous blood was collected in BD Vacutainers (BD, San Jose, CA) containing EDTA from participating family members and controls. Genomic DNA was extracted with QIAamp DNA Blood Mini Kits (Qiagen Science, Germantown, MD).

### Mutation detection

All coding exons and intron-exon junction of the candidate genes known to be associated with congenital cataract, including *CRYAA, CRYAB, CRYBA1, CRYBB1, CRYBB2, CRYGC, CRYGD, CRYGS, GJA3, GJA8, MIP, HSF4,* and *BFSP2*, were amplified with PCR with the primers listed in Table 1. Each reaction mix (25 μl) contained 20 ng of genomic DNA, 1× PCR buffer, 1.5 mM MgCl2, 0.2 mM mixture of deoxyribonucleoside-5′-triphosphates (dNTPs), 0.5 μM each of forward and reverse primers, and 2.5 U of Taq DNA polymerase (TianGen, Beijing, China). A PCR program was performed for DNA amplifying: 95 °C for 5 min, followed by 35 cycles at 95 °C for 30 s, 57 °C to 63 °C for 30 s (annealing temperature depending on different primer), and 72 °C for 30 s, and a final extension at 72 °C for 10 min. The PCR products were sequenced using ABI 3730 Automated Sequencer (PE Biosystems, Foster City, CA). The sequencing results were analyzed using Chromas 2.33 and compared to the reference sequence in the NCBI database.

**Table 1:** Primers used for PCR

Name	Forward (5′-3′)	Reverse (5′-3′)
CRYAA-1	AGCAGCCTTCTTCATGAGC	CAAGACCAGAGTCCATCG
CRYAA-2	GGCAGGTGACCGAAGCATC	GAAGGCATGGTGCAGGTG
CRYAA-3	GCAGCTTCTCTGGCATGG	GGGAAGCAAAGGAAGACAGA
CRYAB-1	AACCCCTGACATCACCATTC	AAGGACTCTCCCGTCCTAGC
CRYAB-2	CCATCCCATTCCCTTACCTT	GCCTCCAAAGCTGATAGCAC
CRYAB-3	TCTCTCTGCCTCTTTCCTCA	CCTTGGAGCCCTCTAAATCA
CRYBA1–1	GGCAGAGGGAGAGCAGAGTG	CACTAGGCAGGAGAACTGGG
CRYBA1–2	AGTGAGCAGCAGAGCCAGAA	GGTCAGTCACTGCCTTATGG
CRYBA1–3	AAGCACAGAGTCAGACTGAAGT	CCCCTGTCTGAAGGGACCTG
CRYBA1–4	GTACAGCTCTACTGGGATTG	ACTGATGATAAATAGCATGAACG
CRYBA1–5	GAATGATAGCCATAGCACTAG	TACCGATACGTATGAAATCTGA
CRYBA1–6	CATCTCATACCATTGTGTTGAG	GCAAGGTCTCATGCTTGAGG
CRYBB1–1	CCCTGGCTGGGGTTGTTGA	TGCCTATCTGCCTGTCTGTTTCTC
CRYBB1–2	TAGCGGGGTAATGGAGGGTG	AGGATAAGAGTCTGGGGAGGTGG
CRYBB1–3	CCTGCACTGCTGGCTTTTATTTA	TCTCCAGAGCCCAGAACCATG
CRYBB1–4	CCAACTCCAAGGAAACAGGCATA	CCTCCCTACCCACCATCATCTC
CRYBB1–5	TAGACAGCAGTGGTCCCTGGAGA	AGCACTGGGAGACTGTGGAAGG
CRYBB1–6	CCTAGAAAAGGAAACCGAGGCC	AGCGAGGAAGTCACATCCCAGTA
CRYBB2–1	GTTTGGGGCCAGAGGGGAGTGGT	TGGGCTGGGGAGGGACTTTCAGTA
CRYBB2–2	CCTTCAGCATCCTTTGGGTTCTCT	GCAGTTCTAAAAGCTTCATCAGTC
CRYBB2–3	GTAGCCAGGATTCTGCCATAGGAA	GTGCCCTCTGGAGCATTTCATAGT
CRYBB2–4	GGCCCCCTCACCCATACTCA	CTTCCCTCCTGCCTCAACCTAATC
CRYBB2–5	CTTACCCTTGGGAAGTGGCAATGG	TCAAAGACCCACAGCAGACAAGTT
CRYGC-1	TGCATAAAATCCCCTTACCG	CCTCCCTGTAACCCACATTG
CRYGC-2	TGGTTGGACAAATTCTGGAAG	CCCACCCCATTCACTTCTTA
CRYGD-1	CAGCAGCCCTCCTGCTAT	GGGTCCTGACTTGAGGATGT
CRYGD-2	GCTTTTCTTCTCTTTTTATTTCTGG	AAGAAAGACACAAGCAAATCAGT
CRYGS-2	GAAACCATCAATAGCGTCTAAATG	TGAAAAGCGGGTAGGCTAAA
CRYGS-3	AATTAAGCCACCCAGCTCCT	GGGAGTACACAGTCCCCAGA
CRYGS-4	GACCTGCTGGTGATTTCCAT	CACTGTGGCGAGCACTGTAT
GJA3–1	CGGTGTTCATGAGCATTTTC	CTCTTCAGCTGCTCCTCCTC
GJA3–2	GAGGAGGAGCAGCTGAAGAG	AGCGGTGTGCGCATAGTAG
GJA3–3	TCGGGTTCCCACCCTACTAT	TATCTGCTGGTGGGAAGTGC
GJA8–1	CCGCGTTAGCAAAAACAGAT	CCTCCATGCGGACGTAGT
GJA8–2	GCAGATCATCTTCGTCTCCA	GGCCACAGACAACATGAACA
GJA8–3	CCACGGAGAAAACCATCTTC	GAGCGTAGGAAGGCAGTGTC
GJA8–4	TCGAGGAGAAGATCAGCACA	GGCTGCTGGCTTTGCTTAG
MIP-1	GTGAAGGGGTTAAGAGGC	GGAGTCAGGGCAATAGAG
MIP-2,3	CGGGGAAGTCTTGAGGAG	CACGCAGAAGGAAAGCAG
MIP-4	CCACTAAGG TGGCTGGAA	CTCATGCCCCAAAACTCA
HSF4–1	CATCCCATCCAGCCAGCCTTTTC	GGGCATGGGTGTTCACTGACGT
HSF4–2	CCTCGACCCATATCCCCGTAAG	GCAGGAGCAAGGCAGGCAGTC
HSF4–3	GCGGGAATGAGCAAAGAGGAGG	GCCAAGGCAGGAGAGAGGAAGG
HSF4–4	TCCCCAGCCTCGCCATTCT	CCCGGTGAAGGAGTTTCCAGAG
HSF4–5	GCTGGGGCCTGAGGGAG	GGCTTCCATCTTCTCTTCCTTTT
BFSP2 (1a)	AATGCACAAACCCAAATGGT	AGGCCCTGSSGACACT
BFSP2 (1b)	GAGAGGCGAGTGGTAGTGGA	GGCCTCAGCCTACTCACAAC
BFSP2 (2)	TGCAGACAGAGCATTTCCAC	GAGGGGTGTGAGCTGGATAA
BFSP2 (3)	GCTGCAATTGCCTTCATTTT	GGGTAACCTGACCCAACTTCA
BFSP2 (4)	TCTGTGAAGCCTGTGTCTGG	CCCGGCCTCAATTATTCTTT
BFSP2 (5)	ACCCAGGAGGAGGAGGTTGT	GGGAATCCCCTGGAAACTAA
BFSP2 (6)	GGGGAATAGTCCAGGCTACC	ATGGGTGCCTATGTGAGAGGG
BFSP2 (7)	TTGTTCCAAAGGCCAGATTC	CACTCAAGGGAATCCTTCCA

### Bioinformatics analysis

The amino acid sequences of *CRYAA* from various species (humans, rats, and chickens, *Xenopus laevis*, and zebra fish) were obtained from the NCBI GenBank, and conservation analysis was performed with CLC Main Workbench Software (Aarhus, Denmark). The function impact of the mutation was predicted with PolyPhen. The hydrophobicity change was predicted with ProtScale.

## Results

### Clinical evaluation

**Figure 2:**
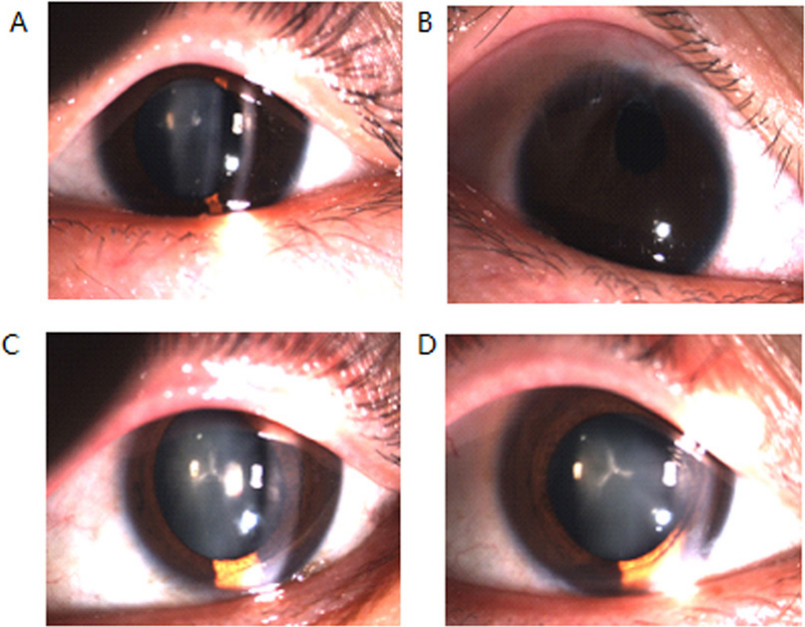
The slit lamp photographs of different individuals. **A**: Slit lamp photographs of the left eye of the proband showed light opacity of Y- suture cataracts with opacities involving nucleus. **B**: Slit lamp photograph of the right eye of the proband which had been extracted showed no opacity. **C** and **D**: The slit-lamp examination of both eyes of the proband's father (II:7) showed Y-suture opacities of the lens involving the nucleus.

All individuals in this family had bilateral cataracts. Slit-lamp examination of the left eye of the proband (III:5), who had been diagnosed with bilateral cataracts at the age of 6 months, revealed slight opacification of Y-suture cataracts, with opacities involving the nucleus (Figure 2A,B). Her right eye had undergone cataract removal when she was aged 14 months, and the best-corrected visual acuity was 0.02/0.6. Slit-lamp examination of both eyes of the proband's father (II:7) showed Y-suture opacities of the lens, involving the nucleus (Figure 2C,D). The best-corrected visual acuity of affected member III:6 was 0.8/0.8. According to the medical records, affected member III:2 underwent cataract removal at the age of 3 years.

### Mutation analysis

**Figure 3:**
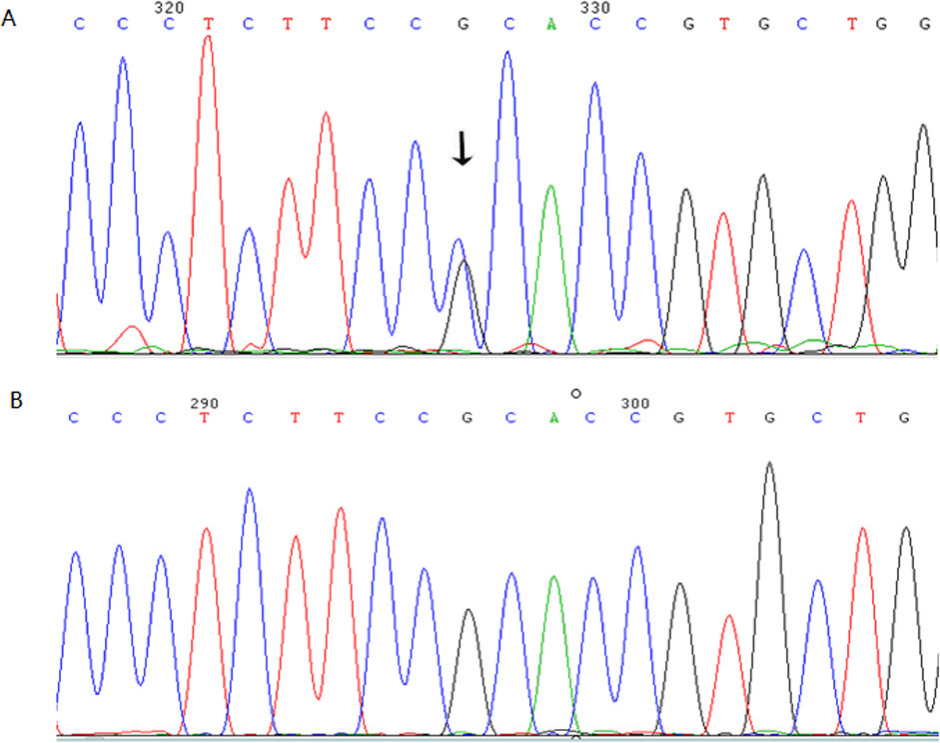
Partial sequence of *CRYAA* at exon1. **A**: The arrow indicates the mutation spot in sequence of affected individual. **B**: No base change was found in the sequence of unaffected individual. In panel **A**, the heterozygous mutation c.161G>C was identified in all the affected participants, but was not found in unaffected family members nor in the 100 unrelated control subjects.

We identified a transversion of G>C at c.161 in exon 1 of crystallin, alpha A (*CRYAA)* in all affected individuals, via direct gene sequencing of the coding regions of the candidate genes (Figure 3). However, we did not find this mutation in any unaffected family members, or in the 100 unrelated control individuals. We found no further gene mutations in individuals from the studied family, except a few nonpathogenic single nucleotide polymorphisms.

### Bioinformatics analysis

**Figure 4:**
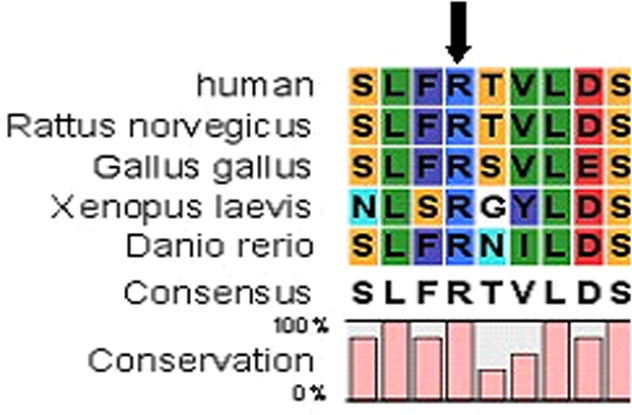
A multiple-sequence alignment of the amino acid sequence in *CRYAA* from different species. The alignment data indicates that the Arg at the 54th amino acid position is highly conserved among many species (indicated by an arrow).

**Figure 5:**
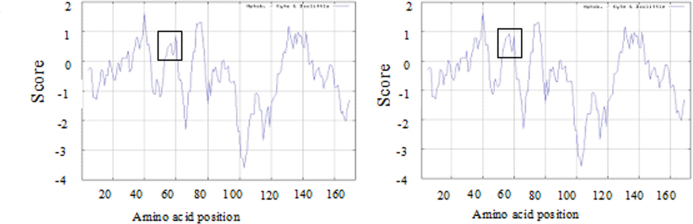
Altered hydrophobicity of R54P in CRYAA protein. **A**: The hydrophobicity of Wild-type CRYAA was predicted using the ProtScale program on the Expasy proteomics server. **B**: The hydrophobicity of mutant type CRYAA was also predicted in the same way. Compared to WT, R54P dramatically enhances the local hydrophobicity, which is indicated by the rectangle.

The c.161G>C mutation resulted in a substitution of arginine (Arg) with praline (Pro) at the 54th amino acid position (R54P). CLC Main Workbench Software revealed that the Arg at the 54th amino acid position is highly conserved among many species (Figure 4). Furthermore, the ProtScale prediction illustrated an increase in local hydrophobicity around the substitution site (Figure 5). In addition, the structure and function impact of the *CRYAA* R54P mutation were predicted by PolyPhen, and the result indicated that R54P could possibly be damaging, with a score of 0.660.

## Discussion

A sutural cataract is defined as an opacity affecting whole or part of the anterior or posterior suture of one or both eyes. Most sutural cataracts are congenital without progression [5], and have been described in association with nuclear, pulverulent, cerulean, and lamellar cataracts [3]. Seven genes have been identified as being associated with sutural cataracts to date (Table2) [5-19], including *BFSP2, CRYBA1/A3, CRYBB1, CRYBB2, GJA8,* and *FTL*. In this study, we identified a novel mutation, c.161G>C, in *CRYAA*, which was associated with isolated Y-suture cataracts in a Chinese family.

**Table 2:** Summary of mutations responsible for Y-suture cataract.

Gene	Sequence change	Lens phenotype	Ethnicity	Reference
*FTL*	32 G>A	Y-suture congenital cataract	Indian	[6]
*GJA8*	235G>C	Full moon with Y-suture cataract	Indian	[7]
*GJA8*	262C>A	Y-suture cataract	Indian	[8]
*MIP*	c.319G>A	one eye exhibited Y-suture and nuclear pulverulent opacification of the lens, while the others exhibited complete opacification	Chinese	[10]
*BFSP2*	697–699delGAA	Y-suture cataract	Chinese	[5]
*BFSP2*	697–699delGAA*	Congenital nuclear and sutural cataract	American	[9]
*BFSP2*	696–698delGAA	Progressive sutural congenital cataract	Chinese	[11]
*BFSP2*	696–698delGAA	Progressive congenital cataract with suture and cortex opacity	Chinese	[12]
*CRYBA1*	IVS3+1G>A	Sutural, nuclear, and peripheral cortical opacity	Australia	[13]
*CRYBA1*	IVS3+1G>C	Zonular and sutural cataract	Brazilian	[14]
*CRYBA1*	IVS3+1G>C	Y-shaped sutural cataract	Saudi Arabian	[15]
*CRYBA1*	IVS3+1 G>A	Progressive childhood cataract with Y-suture opacity	Chinese	[16]
*CRYBA1*	IVS3+1 G>T	Y-suture opacity	Chinese	[17]
*CRYBB2*	475C>T, 483C>T	Sutural cataract and cerulean opacities	Indian	[18]
*CRYBB1*	658G>T	Dustlike cataract with the anterior and posterior Y-suture opacities	American	[19]

CRYAA is the major protein of the eye lens in vertebrates, and plays a structural role in maintaining lens transparency and a proper refractive index. CRYAA is also a member of the small heat-shock-protein (sHSP) family, which are stress-induced proteins, and has chaperone activity [20,21]. *CRYAA* contains a conserved α-crystallin domain (about 90 amino acids), which is flanked on either side by a hydrophobic NH2-terminal domain (about 60 amino acids) and a hydrophilic unstructured COOH-terminal (about 30 amino acids) [22,23]. Genetic and biochemical studies have suggested that some mutant forms of lens opacities or cataracts are associated with decreased chaperone-like activities of α-crystallins. Several mutations in *CRYAA* have been reported to date, and the Arg at the 54th locus of the peptide is a mutation hotspot. In 2007, Arif et al. [24] reported an autosomal recessive congenital total cataract with microcornea caused by a missense mutation of *CRYAA* (R54C). Furthermore, Gong et al. [25] reported that a R54C mutation in *CRYAA* leads to recessive cataracts in humans and mice. In 2008, Devi et al. [26]. showed that a *CRYAA* R54C mutation was associated with autosomal dominant congenital nuclear cataracts associated with microcornea, and a missense *CRYAA* mutation (R54P) was identified in an autosomal dominant family with Y-suture cataract in this study. The 54th amino acid is in the NH2-terminal region of CRYAA, which is an important determinant of α-crystallin aggregate size, and plays a role in the resistance of α-crystallin to environmental stress [27]. In the present study, protein analysis with ProtScale clearly showed an increase in local hydrophobicity around the Arg–Pro substitution site in *CRYAA*. In addition, PolyPhen indicated that R54P is possibly damaging. As exhibited in other lens proteins, hydrophobicity is associated with crystallin activities; increased hydrophobic interaction could reduce their solubility or lead to abnormal folding [28-30]. For CRYAA, changes in hydrophobicity also affect the protein structure and function. Sharna et al. [31] showed that the hydrophobic NH2-terminal domain of the CRYAA protein is involved in chaperone-like activity. Shroff et al. [32] showed that the alpha A-R116C mutant CRYAA led to the generation of a highly oligomerized crystallin, alpha A and decreased chaperone-like function. Thus, we speculate that the increase in local hydrophobicity around the Arg–Pro substitution site in *CRYAA* may affect its oligomerization or chaperoning activity. Furthermore, a knock-in mouse model has demonstrated that the *CRYAA* R49C mutation enhances protein insolubility and cell death [33]. The native protein is an active polypeptide that has antiapoptotic properties that are important for maintaining the survival of lens epithelial cells. Since the 54th amino acid is near the 49th amino acid, the R54P may also have a similar impact; the mutant may be less resistant to environment stress, and enhance cell death. All these changes may have resulted in the cataracts that formed in the studied family.

In conclusion, we are the first to identify that a G→C mutation of *CRYAA* resulted in congenital autosomal dominant Y-suture cataracts. This mutation supports the role of the *CRYAA* gene in human cataract formation and provides further evidence of the genetic heterogeneity of congenital cataracts.
